# Extracting Objective Estimates of Sedentary Behavior from Accelerometer Data: Measurement Considerations for Surveillance and Research Applications

**DOI:** 10.1371/journal.pone.0118078

**Published:** 2015-02-06

**Authors:** Youngdeok Kim, Gregory J. Welk, Saori I. Braun, Minsoo Kang

**Affiliations:** 1 Department of Health, Exercise, and Sport Sciences, Texas Tech University, Lubbock, Texas, United States of America; 2 Department of Kinesiology, Iowa State University, Ames, Iowa, United States of America; 3 Department of Kinesiology, University of Wisconsin-Eau Claire, Eau Claire, Wisconsin, United States of America; 4 Department of Health and Human Performance, Middle Tennessee State University, Murfreesboro, Tennessee, United States of America; School of Public Health of University of São Paulo, BRAZIL

## Abstract

**Background:**

Accelerometer-based activity monitors are widely used in research and surveillance applications for quantifying sedentary behavior (SB) and physical activity (PA). Considerable research has been done to refine methods for assessing PA, but relatively little attention has been given to operationalizing SB parameters (i.e., sedentary time and breaks) from accelerometer data - particularly in relation to health outcomes. This study investigated: (a) the accrued patterns of sedentary time and breaks; and (b) the associations of sedentary time and breaks in different bout durations with cardiovascular risk factors.

**Methods:**

Accelerometer data on 5,917 adults from the National Health Examination and Nutrition Survey (NHANES) 2003–2006 were used. Sedentary time and breaks at different bout durations (i.e., 1, 2–4, 5–9, 10–14, 15–19, 20–24, 25–29, and ≥30-min) were obtained using a threshold of <100 counts per minute. Sedentary time and breaks were regressed on cardiovascular risk factors (waist circumference, triglyceride, and high-density lipoprotein cholesterol) and body mass index across bout durations.

**Results:**

The results revealed that the majority of sedentary time occurred within relatively short bout durations (≈70% and ≈85% for <5-min and <10-min, respectively). The associations of sedentary time and breaks with health outcomes varied depending on how bout time was defined. Estimates of SB parameters based on bout durations of 5 min or shorter were associated with reduced cardiovascular risk factors while durations longer than 10-min were generally associated with increased risk factors.

**Conclusions:**

The present study demonstrates that the duration of sedentary bouts should be further considered when operationalizing the SB parameters from accelerometer data. The threshold of 5 minutes to define a bout is defensible, but a 10 minute threshold would provide a more conservative estimate to clearly capture the prolonged nature of sedentary behavior. Additional research is needed to determine the relative sensitivity and specificity of these thresholds.

## Introduction

The health benefits of physical activity (PA) are well established [1] but a paradigm shift in PA field is now challenging researchers to think about the independent effects of sedentary behavior (SB). This shift evolved from a rapidly growing body of evidence indicating that SB may contribute to individual health risks even if people are physically active [2,3]. According to Owen et al. [4], SB refers to “… *behaviours for which energy expenditure is low*, *including prolonged sitting time in transit*, *at work*, *at home and in leisure time” (p*. *82)*. The Sedentary Behavior Research Network has also proposed definitions of SB that capture both posture (i.e., sitting/reclining) and low levels of energy expenditure (1.0 to 1.5 METs) [5]. These distinctions make conceptual sense to characterize SB as being distinct from physically inactive [6,7], but have proven difficult to operationalize and apply in practice.

One challenge in studies employing objective monitors such as the Actigraph is that posture cannot be readily determined. This makes it impossible to distinguish light-intensity PA (e.g., standing still) from SB [8,9]; however, multi-sensor monitors and new Actigraph versions with gyroscopes may enable this in the future. A more fundamental challenge that has received far less attention is how to quantify the duration of SB. Definitions of SB emphasize time spent in “prolonged” sitting time, but most studies to date have captured total sedentary time without consideration to how it was accumulated. For example, one widely used approach to describe sedentary time from Actigraph accelerometer data is to count every *single minute* where activity counts are less than the threshold for SB [10–14]. However, this method clearly does not take the prolonged aspect of the definition of SB into account. The complexity of this issue was first described in the seminal study by Healy et al. [15] that demonstrated that breaks in sedentary time (defined as interruptions in sedentary time) could explain differences in cardio-metabolic and inflammatory risk biomarkers [12,15]. The authors implied that people who engaged in the same overall amount of sedentary time could have experienced distinct health-related issues depending on how their sedentary time was accumulated. This finding sparked considerable interest in the study of SB as it demonstrated that *breaking up prolonged sedentary time* needs to be factored into evaluations of health risk as well as interventions designed to change behavior [6,16]. Unfortunately, there is presently no consensus on the best way to operationalize and quantify breaks in sedentary time from accelerometer data. Healy et al. [15] originally described breaks in sedentary time as an absolute number of transitions from sedentary to active phase; however, this approach has been questioned due to ambiguity of its measurement properties [9,12] and has led the researchers to wonder if it is a pattern of how sedentary time is accumulated or global measure of breaks that matter more for health [17,18]. One study using the National Health and Nutrition Examination Survey (NHANES) 2003–2006 accelerometer data [19] took breaks in SB into account to evaluate associations with metabolic syndrome. The authors operationally defined the sedentary bout as a period of time at least >5 minute with activity counts <100 cpm with 1 allowable minute outside the threshold; however, this definition/criteria has not been empirically tested or evaluated.

The present study fills that gap by examining the impact of sedentary time and breaks captured using different bout durations. The specific purposes were: 1) to examine the accrued patterns of sedentary time and breaks; and 2) to evaluate the sedentary time and breaks in different bout durations in relation to health outcomes, including cardiovascular risk factors. The study is directly responsive to recommendations for continued research on definitions and measurement of SB [20]. The specific focus on examining sedentary bouts of varying duration in relation to health biomarkers can extend our practical understanding of SB [21] and contribute to future research and intervention development.

## Methods

### Survey Data and Study Sample

The data for the present study were obtained from the NHANES 2003–2004 and 2005–2006. The NHANES provides cross-sectional data for a national representative sample of the US civilian non-institutionalized population selected by a complex multistage probability sampling scheme. The survey measured broad areas of health-related outcomes through household interviews and physical examinations at the mobile examination center (MEC). The original survey protocols were approved by the National Center for Health Statistics (NCHS) Research Ethics Review board, and informed consent was obtained from all participants.

Among participants who visited the MEC, all ambulatory participants (≥ 6 years) were eligible for accelerometer measures. The Actigraph accelerometer (model 7164) was used to obtain objective measures of PA. Participants were instructed to wear the accelerometer on their right hip during waking hours across 7 consecutive days, with the exception of when engaging in any water-based activities (e.g., bathing or swimming). Activity counts that represent integrated acceleration information of ambulatory movements were recorded in minute-by-minute intervals. A detailed description of the NHANES data can be found at www.cdc.gov/nchs/nhanes.htm.

A total of 9,151 adults (≥18 years) provided accelerometer data. After excluding those with missing values for one or more of the cardiovascular risk factors [with an exception of fasting sub-component, triglyceride (TG)] or covariates examined in this study, or with insufficient valid accelerometer data (refer to later section), the final sample consisted of 5,917 adults (2,941 male and 2,976 female) which included a fasting sub-sample of 2,663 who provided TG measure. The final sample was demographically different from the excluded sample (n = 3,234) by gender [unweighted *x* ^2^ (1) = 26.97, *p* <.001] and race/ethnicity [unweighted *x* ^2^ (3) = 25.70, *p* <.001] but similar with respect to family income levels [unweighted *x* ^2^ (3) = 0.84, *p* = .840].

### Accelerometer-based SB Measures

An automated SAS macro provided by NCHS was used for quality control and to identify non-wear times. Non-wear time is defined as intervals of at least 60 minutes of 0 activity counts (i.e., no movement), with allowance for up to 2 consecutive minutes of activity counts between 0 and 100 [22]. After removing the non-wear times from accelerometer data, the following SB parameters were extracted for each measurement day using the traditional algorithms as previously described in the literature [15,23]:

1. sedentary time—a minute where activity counts are <100 cpm;
2. sedentary break—a transition point from a sedentary (<100 cpm) to active phase (≥100 cpm);
3. sedentary bout—a duration of continuous sedentary time (i.e., sedentary event);
4. mean intensity—the average activity counts within each sedentary bout.

In addition, because we aimed to explore accrued patterns of sedentary time and breaks, particularly focusing on sedentary bout durations, all SB measures were extracted within the respective bout durations of 1-min, 2–4 min, 5–9 min, 10–14 min, 15–19 min, 20–24 min, 25–29 min, and ≥30-min in addition to total accrued quantities.

All SB measures obtained for each measurement day were then averaged across only valid days (i.e., 10 or more hours of wear time), to represent the average measures of SB per day. Participants with 4 or more valid days are included in the analysis. In addition, because sedentary time or breaks are influenced by accelerometer wear times [23], the least-square adjustment for wear times was made for all SB measures using the residuals obtained from linear regression models where SB measures were regressed on wear times [12,24].

### Cardiovascular Risk Factors and Other Covariates

The primary purpose of this study was to evaluate the impact of different measures of sedentary time on health outcomes after taking sedentary bout durations into account. Three measures of cardiovascular risk factors were examined in this study [TG (mg/dL), waist circumference (WC) (cm), and high-density lipoprotein cholesterol (HDL-C) (mg/dL)] since previous studies with NHANES data have reported significant associations with SB measures [12,19]. In addition, body mass index (BMI) (kg/m^2^) was also obtained as there is still controversy in regards to its relationship with SB measures in the NHANES data [14].

The average time spent in moderate-to-vigorous-intensity PA (MVPA), based on a modified 10-min bout condition (i.e., a minimum of 8 out of 10 consecutive minutes of MVPA), across valid days was obtained from accelerometer data using the threshold of ≥2020 cpm [22]. Demographic characteristics of the participants including age (years), sex, race/ethnicity (Non-Hispanic White, Non-Hispanic Black, Mexican American, and Other Hispanic/other races), and family income (<$15k, $15k-34.9k, $35k-64.9k, ≥$65k) were also used as covariates in the statistical model (See Table 1 for descriptive statistics).

**Table 1:** Descriptive Statistics of Outcome Variables across Demographic Characteristics among US Adults.

	*%* (*SE*)	Waist Circumference (cm)	HDL-C (mg/dL)	BMI (kg/m^2^)	MVPA (mins·day^-1^)	Fasting sub-sample	
						*%* (*SE*)	Triglycerides (mg/dL)
Total	(*n* = 5,917)	95.43 (.40)	54.36 (.32)	27.75 (.17)	7.38 (.37)	(*n* = 2,667)	144.37 (3.35)
Gender							
Male	48.43 (.61)	98.30 (.53)	48.52 (.32)	27.63 (.19)	9.00 (.47)	48.90 (1.02)	158.50 (3.85)
Female	51.57 (.61)	92.73 (.50)	59.85 (.49)	27.87 (.23)	5.86 (.35)	51.70 (1.02)	131.17 (4.29)
Race							
Non-Hispanic White	71.17 (2.23)	96.15 (.48)	54.29 (.36)	27.66 (.20)	7.06 (.44)	73.25 (2.37)	147.15 (4.32)
Non-Hispanic Black	11.93 (1.63)	96.12 (.63)	57.70 (.74)	29.43 (.26)	7.52 (.57)	10.02 (1.33)	112.85 (3.05)
Mexican American	8.34 (1.12)	94.13 (.65)	51.15 (.50)	27.73 (.24)	8.91 (.57)	7.97 (1.18)	160.90 (.8.29)
Other Hispanic/races	8.57 (.84)	89.76 (.99)	53.47 (.91)	26.26 (.43)	8.32 (1.03)	8.76 (1.06)	142.01 (7.60)
Income							
<$15k	13.78 (.91)	93.19 (.85)	53.44 (.84)	27.27 (.37)	8.86 (.88)	10.44 (.71)	150.76 (8.49)
$15k-34.9k	25.33 (1.07)	95.18 (.72)	54.90 (.56)	27.50 (.25)	6.00 (.42)	24.73 (1.10)	149.16 (9.16)
$35k-64.9k	28.21 (1.10)	96.74 (.58)	53.26 (.49)	28.26 (.22)	7.04 (.59)	29.27 (1.39)	145.15 (4.63)
≥$65k	32.69 (1.76)	95.42 (.61)	55.29 (.41)	27.72 (.26)	8.12 (.44)	35.56 (1.96)	138.53 (3.69)

*Note*. All values are the survey-weighted means (standard error) unless otherwise specified

HDL-C = high-density lipoprotein cholesterol; BMI = body mass index; MVPA = moderate-to-vigorous-intensity physical activity

### Statistical Analyses

To explore the accumulation patterns of SB measures, descriptive statistics as well as the proportions (%) of accrued sedentary time and breaks within each bout duration were estimated. Univariate normality of sedentary time and breaks within each bout duration were examined by the skewness and kurtosis statistics in order to assure the use of parameterized linear models for sequential steps.

To evaluate the measurement properties of accrued sedentary time and breaks across a set of bout durations, bivariate correlation analyses with total sedentary time and breaks were performed using a mean of uncorrected item-total correlation analysis. Item-total correlation analysis is a well-known statistical approach for evaluating the construct validity of measurement at the item level [25]. In this study, we assumed that the sedentary time and breaks within each bout duration were the sub-items that consisted of total sedentary time and breaks, respectively. A positive and relatively high correlation coefficient of ≥.40 [25] was expected for each of the correlation analyses. In addition, bivariate correlation analyses for pairs of sedentary time and breaks at each bout duration were conducted as the secondary analysis to aid in better understanding of the practical significance of sedentary breaks after taking bout durations into account.

Lastly, separate linear regression models were fitted for each bout duration in order to evaluate the measurement properties of sedentary time and breaks within each bout duration in relation to cardiovascular risk factors after controlling for covariates. In this analysis, total sedentary time or breaks were not adjusted in each regression model due to the fact that the separate independent associations of the sub-components of total measure with the dependent variable may be unreliably estimated when total measure is adjusted in the model [26,27].

All statistical analyses were performed using SURVEY procedures in SAS v9.3 (SAS Institute Inc, Cary, NC) to account for the complex sampling designs in the NHANES. Four-year sample weights were calculated using the 2-year sample weights of the NHANES 2003–2004 and 2005–2006 cycles. To account for a selection bias by inclusion criteria of this study, 4-year sample weights were recalculated based on sample weights in the NHANES 2003–2006 data after taking age, gender, and racial/ethnic groups into account. For the analyses of fasting sub-component measure (i.e., TG), four-year fasting sub-sample weights were used. A prior significance level was set at *p* <.05 for all statistical analyses.

## Results

Descriptive statistics for accrued SB measures are presented in Table 2. US adults spent an average of 482.88 minutes per day in sedentary time, which were accumulated over an average of 93.02 (SE = .31) sedentary bouts. The majority of sedentary behaviors was observed within bout durations of <10 minutes (Sedentary bout: 1-min = 36.30%, 2–4 min = 33.27%, and 5–9 min = 15.53%). Similarly, of the 92.41 total sedentary breaks, the majority of sedentary breaks were also detected within bout durations of 1-min (36.54%), 2–4 min (33.65%), and 5–9 min (15.56%).

**Table 2:** Accelerometer Determined Sedentary Behavior Measures across the Bout Durations (*n* = 5,917).

	Total	Sedentary bout durations							
		1-min	2–4 min	5–9 min	10–14 min	15–19 min	20–24 min	25–29 min	≥30-min
Sedentary times									
*Mean* (*SE*) min·day^-1^	482.88 (1.93)	34.25 (.17)	84.96 (.41)	94.04 (.33)	62.45 (.34)	44.47 (.28)	33.36 (.28)	25.69 (.26)	103.66 (1.30)
*%* (*SE*)	-	8.12 (.06)	19.21 (.12)	20.19 (.08)	12.89 (.06)	8.91 (.05)	6.58 (.05)	4.95 (.04)	19.16 (.19)
Correlation with total sedentary time ^a^	-	-0.64	-0.24	0.35	0.65	0.70	0.69	0.66	0.77
Sedentary breaks									
*Mean* (*SE*) times·day^-1^	92.41 (.31)	34.06 (.17)	31.43 (.16)	14.35 (.05)	5.30 (.03)	2.62 (.02)	1.50 (.01)	0.93 (.01)	2.21 (.03)
*%* (*SE*)	-	36.54 (.10)	33.65 (.07)	15.56 (.05)	5.87 (.04)	2.95 (.02)	1.72 (.02)	1.08 (.07)	2.62 (.04)
Correlation with total sedentary breaks ^b^	-	0.80	0.94	0.61	0.04	-0.19	-0.33	-0.40	-0.60
Correlation between sedentary time and breaks ^c^	-0.23	1.00	0.99	0.99	1.00	1.00	0.99	0.99	0.97
Sedentary bouts									
*Mean* (*SE*) times·day^-1^	93.02 (.31)	34.25 (.17)	31.53 (.16)	14.41 (.05)	5.34 (.03)	2.65 (.02)	1.53 (.01)	0.96 (.01)	2.35 (.03)
*%* (*SE*)	-	36.30 (.10)	33.27 (.07)	15.53 (.05)	5.96 (.03)	3.04 (.02)	1.80 (.02)	1.15 (.01)	2.96 (.04)
Average intensity during sedentary time									
*Mean* (*SE*) cpm·day^-1^	30.43 (.11)	42.17 (.14)	30.77 (.11)	20.40 (.10)	14.51 (.09)	11.54 (.08)	9.55 (.07)	8.27 (.07)	6.02 (.07)

*Note*. All estimates were adjusted for accelerometer wear time

*SE* = standard error; CPM = counts per minute

^a^ correlation coefficients between sedentary time in each bout duration and total sedentary time

^b^ correlation coefficients between sedentary breaks in each bout duration and total sedentary breaks

^c^ correlation coefficients between the pairs of sedentary time and breaks within each bout duration

Bivariate correlation analyses of sedentary time and bout durations revealed negative relationships for durations of 1-min and 2–4 min (*r =* -.64 and *r =* -.24, respectively) and a positive relationship for durations of 5–9 min (*r =* .35). In contrast, consistently strong positive relationships were found between sedentary breaks and each of the bout duration indicators (1-min: *r =* .80, 2–4 min: *r =* .94, and 5–9 min: *r =* .61).

The results of the regression analyses are presented in Table 3. Overall, total sedentary time was significantly associated with the three cardiovascular risk factors (WC: *b =* .005, *p* = .049; HDL-C: *b =* -.012, *p <*.001; TG: *b =* .113, *p <*.001) but not BMI (*b =* .001; *p =* .521). Separate regression analyses using estimates computed with different bout durations revealed mixed associations with health outcomes. Specifically, sedentary time estimates based on bout durations of 1-min, 2–4 min, or 5–9 min were negatively (favorably) associated with both WC (1-min: *b* = -0.189, *p* <.001; 2–4 min: *b* = -0.083, *p* <.001; 5–9 min: *b* = -0.032, *p* = .003) and BMI (1-min: *b* = -0.056, *p* <.001; 2–4 min: *b* = -0.025, *p* <.001; 5–9 min: *b* = -0.014, *p* = .004). In contrast, estimates of sedentary time based on bout durations of ≥ 10 minutes (e.g. 10–14 min, etc…) yielded consistently positive (unfavorable) associations with most health outcomes (e.g. WC: all p’s <.05).

**Table 3:** Associations of Accelerometer Determined Sedentary Behavior Measures with Health Outcomes across the Bout Durations (*n* = 5,917).

	*Mean* (*SE*)	Waist Circumference (cm)		HDL-C (mg/dL)		Triglyceride^a^ (mg/dL)		BMI (kg/m^2^)	
		*b* (*SE*)	*p*-value	*b* (*SE*)	*p*-value	*b* (*SE*)	*p*-value	*b* (*SE*)	*p*-value
Sedentary time									
Total	482.88 (1.93)	0.005 (.00)	.049^*^	-0.012 (.00)	<.001^*^	0.113 (.03)	<.001^*^	0.001 (.00)	0.521
Bout durations									
1-min	34.25 (.17)	-0.189 (.03)	<.001^*^	0.111 (.03)	<.001^*^	-1.136 (.26)	<.001^*^	-0.056 (.01)	<.001^*^
2–4 min	84.96 (.41)	-0.083 (.02)	<.001^*^	0.021 (.01)	.102	-0.044 (.16)	.786	-0.025 (.01)	<.001^*^
5–9 min	94.04 (.33)	-0.032 (.01)	.003^*^	-0.024 (.01)	.032^*^	0.545 (.25)	.035^*^	-0.014 (.01)	.004^*^
10–14 min	62.45 (.34)	0.034 (.01)	.023^*^	-0.054 (.01)	<.001^*^	0.730 (.20)	.001^*^	0.007 (.01)	.198
15–19 min	44.47 (.28)	0.038 (.01)	.006^*^	-0.050 (.01)	.002^*^	0.440 (.12)	.001^*^	0.009 (.01)	.092
20–24 min	33.36 (.28)	0.040 (.02)	.036^*^	-0.041 (.02)	.010^*^	0.379 (.14)	.009^*^	0.008 (.01)	.229
25–29 min	25.69 (.26)	0.050 (.02)	.009^*^	-0.046 (.02)	.016^*^	0.549 (.15)	.001^*^	0.012 (.01)	.088
≥30-min	103.66 (1.30)	0.013 (.00)	.001^*^	-0.012 (.00)	.007^*^	0.053 (.05)	.282	0.003 (.00)	.043^*^
Sedentary breaks									
Total	92.41 (.31)	-0.124 (.02)	<.001^*^	0.028 (.02)	.141	-0.099 (.23)	.675	-0.040 (.01)	<.001^*^
Bout durations									
1-min	34.06 (.17)	-0.188 (.03)	<.001^*^	0.111 (.03)	<.001^*^	-1.141 (.26)	<.001^*^	-0.056 (.01)	<.001^*^
2–4 min	31.43 (.16)	-0.226 (.04)	<.001^*^	0.066 (.04)	.074	-0.223 (.46)	.632	-0.067 (.02)	<.001^*^
5–9 min	14.35 (.05)	-0.241 (.07)	.002^*^	-0.136 (.07)	.063	3.207 (1.51)	.042^*^	-0.099 (.03)	.002^*^
10–14 min	5.30 (.03)	0.392 (.17)	.025^*^	-0.645 (.14)	<.001^*^	8.723 (2.47)	.001^*^	0.084 (.07)	.213
15–19 min	2.62 (.02)	0.625 (.23)	.001^*^	-0.814 (.25)	.003^*^	7.467 (2.10)	.001^*^	0.140 (.09)	.112
20–24 min	1.50 (.01)	0.894 (.41)	.037^*^	-0.924 (.35)	.012^*^	8.607 (2.92)	.006^*^	0.194 (.15)	.213
25–29 min	0.93 (.01)	1.345 (.51)	.013^*^	-1.337 (.48)	.010^*^	14.137 (4.07)	.002^*^	0.328 (.20)	.106
≥30-min	2.21 (.03)	0.704 (.19)	<.001^*^	-0.641 (.21)	.005^*^	2.806 (2.43)	.257	0.165 (.08)	.033^*^

*Note*. Separate regression analyses were conducted by each bout durations after adjusting for age, sex, race/ethnicity, family income, and MVPA min·day^-1^.

HDL-C = high-density lipoprotein cholesterol; BMI = body mass index

^a^ the estimates were based on the fasting sub-sample of 2,663adults.

^*^ *p*<.05

Sedentary breaks provide an alternate way to examine associations with health outcomes. The total number of sedentary breaks was negatively (favorably) associated with WC (*b* = -0.124, *p* <.001) and BMI (*b* = -0.040, *p* <.001). However, similar to sedentary time, associations between sedentary breaks and health outcomes also varied by bout durations. For instance, significant negative (favorable) associations between sedentary breaks and WC were detected at bout durations of <10–14 min (1-min: *b* = -0.188, *p* <.001; 2–4 min: *b* = -0.226, *p* <.001; 5–9 min: *b* = -0.241, *p* = .002) while opposite (positive) associations were detected at longer bout durations (all *p*’s < .05). Nonsignificant associations were observed between total sedentary breaks and both HDL-C (*b* = 0.028, *p* = .141) and TG (*b* = -0.099, *p* = .675) when sedentary breaks were computed with bout duration of 1-min. However, significant positive correlations were detected with HDL-C (*b* = 0.111, *p* <.001) and TG (*b* = -1.141, *p* <.001) when breaks were computed for longer bout durations.

To extend the understanding of the influence of bout durations on the relationship with health outcomes, we created two sets of composite variables for sedentary time and breaks based on the thresholds of <5-min and <10-min bout durations. The bivariate correlation analyses for pairs of sedentary time and breaks showed relatively high inter-relationships for both lengths (*r*’s = .973 and .772 for 5-min criterion; *r*’s = .883 and .923 for 10-min criterion) (see Table 4). The separate regression analyses using new composite variables showed consistent trends where the implications of the relationships of sedentary time and breaks with health outcomes tended to be differentiated by the thresholds of bout durations (see Table 5). For instance of 5-min criterion, sedentary time at <5-min was associated with decreased level of WC (*b =* -0.068, *p <*.001) while sedentary time at ≥5-min was associated with increased level of WC (*b =* 0.008, *p <* .001).

**Table 4:** Bivariate Correlation Matrix between Sedentary Time and Breaks across Different Bout Duration Conditions (*n* = 5,917).

	Mean (SE)	Sedentary Time			
		5-min criterion		10-min criterion	
		<5-min	≥5-min	<10-min	≥10-min
*Mean* (*SE*)		119.21(.56)	363.67 (2.08)	213.25 (.75)	269.2.02)
Sedentary breaks					
5-min criterion					
<5-min	65.49 (.32)	0.973	-	-	-
≥5-min	26.92 (.10)	-	0.772	-	-
10-min criterion					
<10-min	79.84 (.34)	-	-	0.883	-
≥10-min	12.57 (.07)	-	-	-	0.923

**Table 5:** Associations of Accelerometer Determined Sedentary Behavior Measures with Health Outcomes across Different Bout Duration Conditions (*n* = 5,917).

	Mean (SE)	Waist Circumference (cm)		HDL-C (mg/dL)		Triglyceride^a^ (mg/dL)		BMI (kg/m^2^)	
		*b* (*SE*)	*p*-value	*b* (*SE*)	*p*-value	*b* (*SE*)	*p*-value	*b* (*SE*)	*p*-value
Sedentary time									
Total	482.88 (1.93)	0.005 (.00)	.049^*^	-0.012 (.00)	<.001^*^	0.113 (.03)	<.001^*^	0.001 (.00)	0.521
5-min criterion									
<5-min	119.21 (.56)	-0.068 (.01)	<.001^*^	0.024 (.01)	.019^*^	-0.157 (.11)	.160	-0.020 (.00)	<.001^*^
≥5-min	363.67 (2.08)	0.008 (.00)	.001^*^	-0.011 (.00)	<.001^*^	0.097 (.02)	<.001^*^	0.002 (.00)	.079
10-min criterion									
<10-min	213.25 (.75)	-0.039 (.01)	<.001^*^	0.003 (.01)	.593	-0.096 (.11)	.387	-0.013 (.00)	<.001^*^
≥10-min	269.63 (2.02)	0.010 (.00)	<.001^*^	-0.010 (.00)	<.001^*^	0.081 (.02)	.003^*^	0.002 (.00)	.024^*^
Sedentary breaks									
Total	92.41 (.31)	-0.124 (.02)	<.001^*^	0.028 (.02)	.141	-0.099 (.23)	.675	-0.040 (.01)	<.001^*^
5-min criterion									
<5-min	65.49 (.32)	-0.120 (.02)	<.001^*^	0.055 (.02)	.005^*^	-0.457 (.18)	.015^*^	-0.036 (.01)	<.001^*^
≥5-min	26.92 (.10)	0.044 (.04)	.293	-0.191 (.04)	<.001^*^	2.464 (.61)	<.001^*^	0.005 (.02)	.774
10-min criterion									
<10-min	79.84 (.34)	-0.113 (.02)	<.001^*^	0.041 (.02)	.019^*^	-0.261 (.19)	.176	-0.035 (.01)	<.001^*^
≥10-min	12.57 (.07)	0.250 (.07)	<.001^*^	-0.298 (.07)	<.001^*^	2.872 (.55)	<.001^*^	0.056 (.03)	.041^*^

*Note*. Separate regression analyses were conducted by each bout duration condition after adjusting for age, sex, race/ethnicity, family income, and MVPA min·day^-1^.

HDL-C = high-density lipoprotein cholesterol; BMI = body mass index.

^a^ the estimates were based on the fasting sub-sample of 2,663 adults.

^*^ *P* <.05

## Discussion

Objectively measured SB using accelerometers has been examined in relation to a variety of health outcomes. Although the body of literature has shown promising implications of reducing prolonged sedentary time and increasing sedentary breaks as they may provide potential health benefits, little attention has been given to the issues related to data processing of accelerometer data to operationalize SB parameters. In this study, we found two main issues in converting accelerometer data to SB parameters that are worth discussing.

### How Long is the Minimum Sedentary Bout Duration to Define Prolonged Sedentary Time?

SB has been defined as any activity during waking hours that requires low energy expenditures of <1.5 MET, which typically involves prolonged sitting or a reclined posture such as watching TV, working on a computer, or driving a car [6,7]. However, there has been a lack of clear definition regarding the minimum duration of sedentary bout that could potentially be considered as prolonged sedentary time, requiring more efforts to explore the accrued patterns of sedentary time in relation to health biomarkers.

The descriptive analyses of the accrued patterns of sedentary time showed that the majority of sedentary time occurred within relatively short bout durations. Of an average of 93.02 sedentary bouts, approximately 70% were attributed to the sedentary time that occurred at bout durations of <5-min (≈85% for <10-min), which accounted for approximately 27% of total sedentary time (≈47% for <10-min). Furthermore, bivariate correlations analyses for pairs of sedentary time at bout durations of 1-min, 2–4 min, and 5–9 min with total sedentary time showed negative or relatively weak linear relationships (*r’s* <.40). These findings imply that the sedentary time that lasts over relatively short durations may not represent the same measurement construct as the sedentary time at relatively long bout durations.

To further examine the significance of bout durations in objectively measured sedentary time, separate linear regression analyses were performed in relation to the health outcomes. Pertaining to total sedentary time, our findings are consistent with previous studies [12,28], where significant and unfavorable associations were observed with WC, HDL-C, and TG. Moreover, our result showing the insignificant relationship of total sedentary time with BMI was also consistent with a recent study [14] that used the same study sample. However, the associations between the computed total sedentary time and health outcomes varied depending on how bouts were defined. As shown in Table 5, the accrued sedentary time computed using bout durations of less than 5 minutes yielded negative correlations for most risk factors (WC, HDL-C, and BMI). When sedentary time was computed as more prolonged periods of time (i.e. bout durations ≥10 minutes), however, the associations were reversed.

In evaluating these findings it is important to consider the capabilities of the Actigraph for detecting sedentary time. The Actigraph accelerometer (model 7164) used in NHANES is an energy classification device that records the accelerations of vertical movements of the waist in forms of activity counts [29]. There have been different thresholds proposed to identify SB (e.g., <150 cpm [8], or <50 cpm [30]) for waist-mounted Actigraph accelerometers, of which an activity threshold of <100 cpm has broadly been used for classifying sedentary time and this has been supported in a number of past studies [23,31]. Regardless of the threshold, however, it is important to acknowledge that the Actigraph accelerometer based on a single vertical axis is not able to distinguish changes in posture (e.g., sitting vs. standing) [8,29]. Thus, epochs that feature activity counts <100 cpm may also include some light-intensity PAs in a standing position, such as washing dishes or folding laundry [32], which can possibly produce positive physiological effects by the postural muscle activations [3]. The simple accumulation of minutes with activity counts <100 cpm would therefore lead to overestimations of sedentary time [33], which in turn influence the implication of the relationship between SB and health outcomes. The constraint of tracking minutes only if they were accumulated for 5 or longer consecutive minutes would help to avoid capturing intermittent light-intensity PAs. Bankoski et al. [19] suggested a duration of >5 minutes be used to distinguish bouts of sedentary time and these analyses support this threshold. However, the selection of any threshold of minimum bout duration would lead to tradeoffs between sensitivity and specificity in the ability to accurately identify periods of SB. The decision between a duration of 5 or longer minutes would depend on the relative importance of false positives or false negatives in the analyses and future study is warranted for further investigation on this issue.

Using shorter epochs or intervals in summarizing accelerometer data would provide better descriptions of the continuity of human movement in a free-living environment. However, the duration to define a bout of SB must be specified to ensure that the accumulated minutes are congruent with the conceptual definition (i.e., the time spent in prolonged SB). The major context of SB (e.g., watching TV, driving a car, etc.) is predominated by prolonged sitting with low energy expenditure [6]. Relying on a single minute or shorter bout to estimate the time spent in SB from accelerometer data may inappropriately include time spent in *sporadic sedentary behavior* but not necessarily *prolonged sedentary behavior*. For example, a person may take brief breaks in a sitting position during an exercise session but this time should not be considered as part of the accumulated time spent in *prolonged sedentary behavior*. This may be an issue not only for Actigraph accelerometers but also for other accelerometers such as the ActivPAL (PAL Technologies Ltd, Glasgow, UK) which directly evaluate posture. A recent study with the ActivPAL in adolescents [34] defined a 15-second interval as a minimum duration of sitting time and the results also highlighted that a large number of sitting events occurred in short bout durations of <5-min. Although it would be challenging to determine the minimum duration of sedentary time which may negatively impact the physiological responses in the human body, efforts are needed to distinguish the time spent in *prolonged sedentary behavior* from accelerometer data should be made when the implication of the public health message is placed on *reducing prolonged sedentary time* and not on total sedentary time.

### Are We Measuring Sedentary Breaks or the Number of Sedentary Bouts?

Promoting breaks in sedentary behavior has emerged as a promising intervention strategy to promote metabolic fitness and help decrease the risk of cardiovascular diseases [6,15]. Dunstan et al. [35] highlighted that even short bout of interruptions in sedentary time with light- or moderate-intensity walking significantly reduced the levels of postprandial glucose and insulin. Despite its potential for improving public health, little is known as to how to operationalize the breaks in sedentary time from accelerometer data that may provide the outputs congruent with what has been defined in the conceptual level. Particularly, the absolute number of transitions from sedentary to active phase has been extensively examined assuming that sedentary time is considered to be interrupted when transition occurs [15]; however, the measurement properties of this approach has been questioned.

In this study, the accrued patterns of sedentary breaks using the algorithm used by Healy et al. [15] showed that approximately 70% of sedentary breaks occurred at sedentary bouts of <5-min. (85% for <10-min), which is almost identical to what we found with respect to the number of sedentary bouts. Furthermore, the accrued sedentary breaks at 1-min bout duration are identical to the accrued sedentary time at 1-min bout duration. The underlying reason for this finding is that the current algorithm to extract sedentary breaks from accelerometer data is, indeed, an alternative expression to extract the number of sedentary bouts. In other words, counting the number of transition points from a sedentary to active phase would produce the same or slightly smaller quantity as the number of sedentary bouts. Only small differences would exist depending on the existence of sedentary time at the end of a continuous measurement period (e.g., sedentary time at the end of wear time or day would be considered as a sedentary bout but not counted for sedentary breaks). This notion could also be supported by the perfect linear relationships between sedentary time and breaks at each bout duration which clearly imply that sedentary breaks obtained by the current algorithm are an alternative parameter that quantifies the amount of sedentary time at each bout duration.

Meanwhile, the correlation coefficient between total sedentary time and total sedentary breaks was -.23 (See Table 2), which may lead to the conclusion of a weak relationship between total sedentary time and breaks [12]. However, this is because of the expansion of range in total sedentary time that significantly attenuates the relationship with total sedentary breaks. For example, 1-unit increases in total sedentary breaks may indicate an increase in total sedentary time by a minimum of one to thirty minutes or more depending on the sedentary bout durations where the breaks occurred. As shown in Table 4, the correlation coefficients between sedentary time and breaks get close to 1.0 as the range of sedentary time narrows for 1-unit increases in sedentary breaks.

While acknowledging the operational limitations of breaks in sedentary time, the transitions from a sedentary to active phase may include standing from a sitting position or walking a step which could also be considered as an indicator of PA. [15]. As illustrated in Table 5, our results showed that favorable associations with health outcomes were consistently found when bouts were defined as being <5 min. However, unfavorable associations were observed when SB was defined using bout durations of ≥10-min. These findings are likely similar to the results we found with respect to sedentary time at each bout duration. Given that the sedentary breaks is an alternative score that represents the number of sedentary bouts as discussed above, the implications may be more related to the patterns of how sedentary time is accumulated rather than to PA which may matter for health (e.g., a higher number of sedentary events with long bout is negatively associated with health outcome). Therefore, sedentary breaks presented as absolute number of transitions from sedentary to active phase may not be considered as breaks in sedentary time or an indicator of PA, and caution is warranted when drawing conclusions about sedentary breaks in relation to health outcome.

Taken together all above mentioned evidence, it is plausible to say that sedentary breaks represented by absolute number of transitions from sedentary to active phase is an incomplete measure of the patterns of sedentary time that does not take account for the respective bout durations which may matter more for health outcomes. Lyden et al. [9] proposed break rate calculated by total number of breaks divided by total sedentary time as a feasible metric specifically for detecting intervention effects. However, given that the breaks is an alternative measure of number of sedentary bouts, it also represents the average number of sedentary bouts to accumulate one sedentary hour. This may be more relevant to the accumulation patterns of sedentary time rather than to breaks in sedentary time, and more efforts to distinguish the breaks in sedentary time from the patterns of sedentary time accumulation should be made.

We believe that identifying the breaks or interruption in sedentary time in the observational study is a difficult task that cannot be comparable to examining the patterns of how sedentary time is accumulated. The noun break refers to *the interruption of continuity or uniformity* or *a pause in work or during an activity or event* [36]. This may imply that true breaks in sedentary time would possibly exist only within a continuous bout of SB pursuit. In other words, a strong assumption has to be made that being sedentary is a fundamental behavior of the participants during the measurement period if the transition from a sedentary to active phase is to be considered as a sedentary break. For example, in the laboratory experimental study conducted by Dunstan et al. [35], the participants were instructed to sit over 7 hours beginning with 2 hours to achieve steady state and then trial conditions (interruptions by 2-minute bouts of walking activities) were applied during the remaining 5 hours. The trial protocols were well-designed to fully reflect the conceptual definition of sedentary breaks (breaks or interruptions in sedentary time) because the participants were, again, forced to be sedentary during the measurement period. However, from an evolutionary perspective, humans are born to be active [37], and it may not be legitimate to consider a simple transition from a sedentary to active as a “break in sedentary time” in a free-living environment in which we do not know whether the observed transitions have purposely occurred within the continuity of SB pursuits or just simply at the true end of SB pursuit. One possible approach to identify objectively measured sedentary breaks in free-living environments could be a combination with subjective measures (e.g., PA log/diary) [38], from which one can obtain the time period information where the fundamental behavior of the participants were expected or assumed to be sedentary (e.g., office hours).

There have been a few attempts to operationally define the *sedentary behavior bout* that may possibly include true breaks within the bout. Carson and Janssen [39] defined a SB bout as a period of ≥30-minutes in which ≥80% of minutes are sedentary (i.e., <100 cpm) with no more than 5 consecutive minutes ≥100 cpm, from which the number of transitions from a sedentary to active phase was then extracted. Although there could be some practical issues such as restricting the break durations to <5 minutes, this could be one possible approach to overcome the limitation that may distinguish the operationalization of sedentary breaks from the patterns of sedentary time accumulation.

This study is not without limitations. First, the final analytic sample was demographically different from the sample excluded by selection criteria. Although the sampling weights were recalculated in order to produce unbiased estimates of population parameters of US adults, such differences may influence the precision of estimates due to the possible loss of non-ignorable missing data in the excluded sample. Second, the present study is data-driven research that relies on cross-sectional data that limits our ability to draw causal relationships of SB measures with health outcomes. Moreover, the implications of our findings are based on the practice of Actigraph accelerometer used in the NHANES 2003–2006 cycles, and caution is needed when interpreting the results for the study with different measurement protocols compared to the NHANES. Finally, the main focus of this study was limited to the data processing issues, particularly focusing on SB measures. There are several important issues in using accelerometer data for a large observational study, such as participants’ compliance, non-wear time, or defining non-wear time which could not be addressed in the present study. The readers who are interested in these particular issues should refer to previous research [20,40–42] for an extensive understanding of those issues and to potentially address them in future research.

SB is purposefully engaged activities in different contexts that are predominated by prolonged sedentary time with low energy expenditure and possibly include sedentary breaks [6]. However, the most commonly used algorithms to obtain SB parameters from accelerometer data may not perform well enough to fully reflect the conceptual definitions of respective parameters in free-living settings. The present study elucidated the necessity of determining the minimum duration of sedentary time that can potentially be considered as prolonged SB. Prior information on SB bouts would be required in order to identify true sedentary breaks that fully reflect the conceptual definition of sedentary breaks (i.e., interruptions in sedentary time). Future research should be aimed at developing a new algorithm/approach to discriminate SB bouts from accelerometer data in order to improve the measurement properties of objectively measured SB in health outcome research.

## References

[1] Physical Activity Guidelines Advisory Committee (2008) Physical Activity Guidelines Advisory Committee Report, 2008. Washington, DC: US Department of Health and Human Services 2008 (2008): A1-H14.

[2] Owen N, Leslie E, Salmon J, Fotheringham MJ (2000) Environmental determinants of physical activity and sedentary behavior. Exercise and Sport Sciences Reviews 28: 153–158. 11064848

[3] Hamilton MT, Healy GN, Dunstan DW, Zderic TW, Owen N (2008) Too little exercise and too much sitting: inactivity physiology and the need for new recommendations on sedentary behavior. Current Cardiovascular Risk Reports 2: 292–298. 22905272 10.1007/s12170-008-0054-8 PMC3419586

[4] Owen N, Bauman A, Brown W (2009) Too much sitting: A novel and important predictor of chronic disease risk? British Journal of Sports Medicine 43: 81–83. 10.1136/bjsm.2008.055269 19050003

[5] Sedentary Behavior Research Network (2012) Letter to the Editor: Standardized use of the terms of "sedentary" and "sedentary behaviors". Applied Physiology, Nutrition, and Metabolism 37: 540–542. 10.1139/h2012-024 22540258

[6] Owen N, Healy GN, Matthews CE, Dunstan DW (2010) Too much sitting: The population-health science of sedentary behavior. Exercise and Sport Sciences Reviews 38: 105–113. 10.1097/JES.0b013e3181e373a2 20577058 PMC3404815

[7] Pate RR, O'Neill JR, Lobelo F (2008) The evolving definition of "Sedentary". Exercise and Sport Sciences Reviews 36: 173–178. 10.1097/JES.0b013e3181877d1a 18815485

[8] Kozey-Keadle S, Libertine A, Lyden K, Staudenmayer J, Freedson PS (2011) Validation of wearable monitors for assessing sedentary behavior. Medicine and Science in Sports and Exercise 43: 1561–1567. 10.1249/MSS.0b013e31820ce174 21233777

[9] Lyden K, Kozey-Keadle S, Staudenmayer J, Freedson PS (2012) Validity of two wearable monitors to estimate breaks from sedentary time. Medicine and Science in Sports and Exercise 44: 2243–2252. 10.1249/MSS.0b013e318260c477 22648343 PMC3475768

[10] Lynch BM, Dunstan DW, Healy GN, Winkler E, Eakin E, et al (2010) Objectively measured physical activity and sedentary time of breast cancer survivors, and associations with adiposity: Findings from NHANES (2003–2006). Cancer Causes Control 21: 283–288. 10.1007/s10552-009-9460-6 19882359

[11] Lynch BM, Dunstan DW, Winkler E, Healy GN, Eakin E, et al (2011) Objectively assessed physical activity, sedentary time and waist circumference among prostate cancer survivors: Findings from the National Health and Nutrition Examination Survey (2003–2006). European Journal of Cancer Care 20: 514–519. 10.1111/j.1365-2354.2010.01205.x 20597954

[12] Healy GN, Matthews CE, Dunstan DW, Winkler EA, Owen N (2011) Sedentary time and cardio-metabolic biomarkers in US adults: NHANES 2003–06. European Heart Journal 32: 590–597. 10.1093/eurheartj/ehq451 21224291 PMC3634159

[13] Clark BK, Healy GN, Winkler EA, Gardiner PA, Sugiyama T, et al (2011) Relationship of television time with accelerometer-derived sedentary time: NHANES. Medicine and Science in Sports and Exercise 43: 822–828. 10.1249/MSS.0b013e3182019510 20980928 PMC8477752

[14] Maher CA, Mire E, Harrington DM, Staiano AE, Katzmarzyk PT (2013) The independent and combined associations of physical activity and sedentary behavior with obesity in adults: NHANES 2003‐06. Obesity. 10.1002/oby.20430 PMC4097108 23512825

[15] Healy GN, Dunstan DW, Salmon J, Cerin E, Shaw JE, et al (2008) Breaks in sedentary time beneficial associations with metabolic risk. Diabetes Care 31: 661–666. 10.2337/dc07-2046 18252901

[16] Dunstan DW, Healy GN, Sugiyama T, Owen N (2010) ‘Too Much Sitting’and metabolic risk—has modern technology caught up with us? European Endocrinology 6: 19–23.

[17] Colley RC, Garriguet D, Janssen I, Wong SL, Saunders TJ, et al (2013) The association between accelerometer-measured patterns of sedentary time and health risk in children and youth: results from the Canadian Health Measures Survey. BMC Public Health 13: 200 10.1186/1471-2458-13-200 23497190 PMC3599834

[18] Saunders TJ, Tremblay MS, Mathieu M, Henderson M, O’Loughlin J, et al (2013) Associations of sedentary behavior, sedentary bouts and breaks in sedentary time with cardiometabolic risk in children with a family history of obesity. PloS ONE 8: e79143 10.1371/journal.pone.0079143 24278117 PMC3835898

[19] Bankoski A, Harris TB, McClain JJ, Brychta RJ, Caserotti P, et al (2011) Sedentary activity associated with metabolic syndrome independent of physical activity. Diabetes Care 34: 497–503. 10.2337/dc10-0987 21270206 PMC3024375

[20] Matthews CE, Hagströmer M, Pober DM, Bowles HR (2012) Best practices for using physical activity monitors in population-based research. Medicine and Science in Sports and Exercise 44: S68–S76. 10.1249/MSS.0b013e3182399e5b 22157777 PMC3543867

[21] Owen N (2012) Ambulatory monitoring and sedentary behaviour: a population-health perspective. Physiological Measurement 33: 1801–1810. 10.1088/0967-3334/33/11/1801 23110918

[22] Troiano RP, Berrigan D, Dodd KW, Mâsse LC, Tilert T, et al (2008) Physical activity in the United States measured by accelerometer. Medicine and Science in Sports and Exercise 40: 181–188. 18091006 10.1249/mss.0b013e31815a51b3

[23] Matthews CE, Chen KY, Freedson PS, Buchowski MS, Beech BM, et al (2008) Amount of time spent in sedentary behaviors in the United States, 2003–2004. American Journal of Epidemiology 167: 875–881. 10.1093/aje/kwm390 18303006 PMC3527832

[24] Willett WC, Howe GR, Kushi LH (1997) Adjustment for total energy intake in epidemiologic studies. The American Journal of Clinical Nutrition 65: S1220–S1228. 10.1093/ajcn/65.4.1220S 9094926

[25] Nunnally JC, Bernstein IH, Berge JMF (1967) Psychometric Theory: McGraw-Hill New York

[26] Satia-Abouta J, Galanko JA, Potter JD, Ammerman A, Martin CF, et al (2003) Associations of total energy and macronutrients with colon cancer risk in African Americans and Whites: Results from the North Carolina colon cancer study. American Journal of Epidemiology 158: 951–962. 14607803 10.1093/aje/kwg248

[27] Wacholder S, Schatzkin A, Freedman LS, Kipnis V, Hartman A, et al (1994) Can energy adjustment separate the effects of energy from those of specific macronutrients? American Journal of Epidemiology 140: 848–855. 7977295 10.1093/oxfordjournals.aje.a117333

[28] Henson J, Yates T, Biddle SJH, Edwardson CL, Khunti K, et al. (2013) Associations of objectively measured sedentary behaviour and physical activity with markers of cardiometabolic health. Diabetologia: 1–9. 10.1007/s00125-013-2845-9 23456209

[29] Granat MH (2012) Event-based analysis of free-living behaviour. Physiological Measurement 33: 1785–1800. 10.1088/0967-3334/33/11/1785 23110873

[30] Crouter SE, Dellavalle DM, Haas JD, Frongillo EA, Bassett DR (2013) Validity of actiGraph 2-regression model and Matthews and NHANES and cut-points for assessing free-Living physical activity. Journal of Physical Activity and Health 10: 504–514. 22975460 10.1123/jpah.10.4.504 PMC4199088

[31] Kim Y, Lee J-M, Peters BP, Gaesser GA, Welk GJ (2014) Examination of Different Accelerometer Cut-Points for Assessing Sedentary Behaviors in Children. PloS One 9: e90630 10.1371/journal.pone.0090630 24699259 PMC3974658

[32] Kozey SL, Lyden K, Howe CA, Staudenmayer JW, Freedson PS (2010) Accelerometer output and MET values of common physical activities. Medicine and Science in Sports and Exercise 42: 1776–1784. 10.1249/MSS.0b013e3181d479f2 20142781 PMC2924952

[33] Carr LJ, Mahar MT (2011) Accuracy of intensity and inclinometer output of three activity monitors for identification of sedentary behavior and light-intensity activity. Journal of Obesity 2012. 10.1155/2012/460271 PMC3228344 22175006

[34] Harrington DM, Dowd KP, Bourke AK, Donnelly AE (2011) Cross-sectional analysis of levels and patterns of objectively measured sedentary time in adolescent females. International Journal of Behavioral Nutrition and Physical Activity 8: 120 10.1186/1479-5868-8-120 22035260 PMC3217883

[35] Dunstan DW, Kingwell BA, Larsen R, Healy GN, Cerin E, et al (2012) Breaking up prolonged sitting reduces postprandial glucose and insulin responses. Diabetes Care 35: 976–983. 10.2337/dc11-1931 22374636 PMC3329818

[36] Oxford Dictionaries Online (2013) New York: Oxford University Press Retrived from http://www.oxforddictionaries.com/us/definition/american_english/break. 10.1007/s13197-013-0993-z

[37] Cordain L, Gotshall RW, Eaton SB (1998) Physical activity, energy expenditure and fitness: an evolutionary perspective. International Journal of Sports Medicine 19: 328–335. 9721056 10.1055/s-2007-971926

[38] Healy GN, Clark BK, Winkler EAH, Gardiner PA, Brown WJ, et al (2011) Measurement of adults' sedentary time in population-based studies. American Journal of Preventive Medicine 41: 216–227. 10.1016/j.amepre.2011.05.005 21767730 PMC3179387

[39] Carson V, Janssen I (2011) Volume, patterns, and types of sedentary behavior and cardio-metabolic health in children and adolescents: A cross-sectional study. BMC Public Health 11: 274 10.1186/1471-2458-11-274 21542910 PMC3112118

[40] Tudor-Locke C, Camhi SM, Troiano RP (2012) A catalog of rules, variables, and definitions applied to accelerometer data in the National Health and Nutrition Examination Survey, 2003–2006. Preventing Chronic Disease 9: 11_0332. 10.5888/pcd9.110332 PMC3457743 22698174

[41] Ward DS, Evenson KR, Vaughn A, Rodgers AB, Troiano RP (2005) Accelerometer use in physical activity: Best practices and research recommendations. Medicine and Science in Sports and Exercise 37: S582–S588. 16294121 10.1249/01.mss.0000185292.71933.91

[42] Winkler EAH, Gardiner PA, Clark BK, Matthews CE, Owen N, et al (2012) Identifying sedentary time using automated estimates of accelerometer wear time. British Journal of Sports Medicine 46: 436–442. 10.1136/bjsm.2010.079699 21504965 PMC3534985

